# Effect of EDTA, NaOCl, and HEDP-based irrigants on the mechanical properties of heat treated NiTi endodontic instruments

**DOI:** 10.1038/s41405-025-00371-3

**Published:** 2025-10-21

**Authors:** Jeanne Davril, Rémy Balthazard, Romain Hocquel, Alexandre Reynaud, Éric Mortier, Marin Vincent

**Affiliations:** 1 Départment de dentisterie restauratrice et endodontie, Faculté d’odontologie de Lorraine, Université de Lorraine, Nancy, France; 2 Université de Lorraine, CNRS, Arts et Métiers Institute of Technology, LEM3, Metz, France; 3 CNRS, IJL, Université de Lorraine, Nancy, France; 4 Faculté d’odontologie de Lorraine, Université de Lorraine, Nancy, France; 5 IICT, School of Engineering and Management Vaud, HES-SO University of Applied Sciences and Arts Western, Yverdon-lès-Bains, Switzerland; 6 Université de Lorraine, CNRS, Arts et Métiers Institute of Technology, LEM3, Nancy, France

**Keywords:** Endodontic files, Root canal treatment

## Abstract

**Aims:**

This study evaluated the influence of different root canal irrigants, 3% sodium hypochlorite (NaOCl), 17% ethylenediaminetetraacetic acid (EDTA), and a NaOCl-HEDP combination (Dual Rinse), on the mechanical behavior of nickel-titanium (NiTi) endodontic instruments with identical geometry but varying heat treatments.

**Method:**

A total of 720 One Curve NiTi files (MicroMega, Besançon, France) with three heat treatments (none, C.Wire, S.Wire) were allocated into subgroups exposed to four irrigants (distilled water, NaOCl, NaOCl-HEDP, EDTA) at 21 °C and 35 °C for 1, 5, or 10 min. Mechanical tests were conducted in accordance with ISO 3630-1 to assess bending resistance, maximum torsional resistance, and angular deflection at fracture. Profilometric analysis was performed to identify potential signs of corrosion.

**Results:**

No significant differences were found in maximum bending or torsional torque across irrigant groups. However, EDTA exposure resulted in increased angular deflection at fracture, followed by distilled water. NaOCl showed the lowest angular deflection, while NaOCl-HEDP exhibited intermediate behavior. Heat-treated instruments, particularly S.Wire, consistently showed superior mechanical performance across all test conditions.

## Introduction

In endodontics, two types of irrigating solutions are essential for effective intracanal disinfection: a disinfecting agent, typically sodium hypochlorite (NaOCl), and a chelating agent such as ethylenediaminetetraacetic acid (EDTA), which facilitates the removal of the smear layer and dissolves the inorganic components of dentin. These two solutions are used within the same canal space but cannot be mixed due to chemical interactions that compromise the antimicrobial efficacy of sodium hypochlorite [1].

In recent years, Medcem GmbH (Weinfelden, Switzerland) has introduced a combined irrigant known as Dual Rinse HEDP (1-hydroxyethylidene-1,1-diphosphonic acid). The formulation integrates NaOCl with editronic acid (HEDP) to achieve a dual action: the proteolytic and tissue-dissolving capacity of sodium hypochlorite alongside the continuous chelating effect of HEDP.

HEDP is less aggressive than EDTA [2], allowing it to be used concurrently with disinfecting agents without compromising their efficacy [3, 4]. Numerous studies have demonstrated that this “continuous chelation” approach using the NaOCl-HEDP combination yields equivalent or superior results to the conventional “sequential chelation” method, which involves irrigation with NaOCl followed by 17% EDTA. Continuous chelation has been shown to be effective in canal cleaning and disinfection [5–17], enhancing the dentinal penetration and the bond strength of bioceramic and resin-based sealers [14, 18–26] and preserving dentin’s mechanical properties [5, 6, 14, 27, 28].

Chemical disinfection occurs throughout the endodontic procedure from the access cavity to the final irrigation protocol following the root canal shaping. Therefore, NiTi instruments are continuously exposed to irrigants during shaping, leading to surface corrosion and degradation [29]. These effects can lead to unexpected instrument separation during clinical procedures [30–33].

The impact of NaOCl and EDTA solutions on the mechanical behavior of NiTi endodontic instruments has already been investigated. These studies have primarily focused on surface properties [30, 34–40], as well as on mechanical resistance [32, 41–51]. Very few studies have investigated the impact of HEDP on the mechanical properties of endodontic instruments [52, 53]. The literature remains inconclusive regarding the influence of sodium hypochlorite and EDTA on the mechanical properties of endodontic instruments under clinical conditions. While some studies have reported deleterious effects [30, 36–38, 41], others have found no significant changes in mechanical behavior [43, 45, 50]. Nevertheless, to date, no study has assessed the mechanical effects on instruments subjected simultaneously or sequentially to multiple irrigating solutions, which better reflects clinical practice. Consequently, it is clinically relevant to further examine the “continuous chelation” approach about this topic. Furthermore, the present study seeks to fill an existing gap in the literature concerning the irrigating solutions on endodontic instruments sharing identical geometric design but different heat treatment.

The objectives of the present study were (i) to evaluate the effects of EDTA, NaOCl, and Dual Rinse HEDP solutions on the mechanical properties of thermally treated NiTi endodontic instruments and (ii) to assess the performance of various heat treatments when exposed to these different irrigation solutions.

## Materials and methods

### Experimental groups

For the bending resistance analysis, three groups of 120 NiTi endodontic instruments with identical One Curve (MicroMega, Besançon, France) geometry were utilized. Group 1 included instruments without any heat treatment (austenitic phase), Group 2 comprised instruments with C.Wire heat treatment (hybrid phase), and Group 3 consisted of instruments with S.Wire heat treatment (martensitic phase). The C.Wire heat treatment enables the instrument to exhibit superelastic properties as well as initial shape memory characteristics, due to its high martensite content (hybrid phase). In contrast, S.Wire produces a fully martensitic instrument, displaying exclusively shape memory properties (martensitic phase).

Each group of 120 instruments was further divided into four subgroups of 30 instruments, based on the irrigant to which they were exposed:

- Subgroup 1: distilled water (Phebus, Reims, France; Ref: 1775040—Batch: 00137).
- Subgroup 2: 3% NaOCl (CanalPro, COLTENE, Alstätten, Switzerland; Ref: 65002866—Batch: M8253).
- Subgroup 3: NaOCl-HEDP solution (Dual Rinse, Medcem GmbH, Weinfelden, Switzerland; Ref: 091224DR—Batch: DR241212)—1 part of HEDP per 10 mL of NaOCl, according to the manufacturer’s recommendations.
- Subgroup 4: 17% EDTA (CanalPro, COLTENE, Alstätten, Switzerland; Ref: 6001 9651—Batch: M8085).

Within each subgroup, five instruments were tested after immersion for 1, 5, or 10 min at either 21 °C (room temperature) or 35 °C (intracanal temperature) (Table 1). Immersion times of 5 and 10 min were selected to conform to existing protocols reported in the literature [54]. However, the shorter duration of 1 min was included to better reflect clinical practice.

**Table 1:** Distribution of Experimental Groups.

Groups	Heat treatment	Subgroups	Immersion time and temperature conditions	Sample size
Bending	None	Distilled water 3% NaOCl NaOCl-HEDP 17% EDTA	1 min, 21 °C 1 min, 35 °C 5 min, 21 °C 5 min, 35 °C 10 min, 21 °C 10 min, 35 °C	30 per subgroup (5 per condition)
	C.Wire			
	S.Wire			
Torsion	None			
	C.Wire			
	S.Wire			

Torsional resistance analysis followed the same grouping and exposure protocol (Table 1). In total, 720 instruments were included in this study.

Each bending test was conducted in accordance with ISO 3630-1 and recorded the maximum torque. Each torsion test was also performed following ISO 3630-1 and measured both the maximum torque at fracture and the maximum angular deflection at fracture.

To avoid galvanic corrosion related to the handle [31, 32, 34, 45], the 720 instruments were not fitted with handles. This choice was made to (i) avoid interference with visual analyses and prevent corrosion results originating from the handle rather than the file, and (ii) allow bending and torsion testing in accordance with ISO 3630-1.

By convention, for the remainder of the study, the groups will be identified as follows: X(a,b,c,d)

where:

- X = type of performed test, with “B” for maximum torque during the bending test, “Tf” for maximum torque at fracture during the torsional test, and “Ta” for maximum angular deflection at fracture during the torsional test;
- a = canal irrigant, with 0 = distilled water, 1 = 3% NaOCl, 2 = NaOCl-HEDP, and 3 = 17% EDTA;
- b = temperature, with 0 = 21 °C and 1 = 35 °C;
- c = heat treatment, with 0 = none, 1 = C.Wire, and 2 = S.Wire;
- d = exposure time, with 0 = 1 min, 1 = 5 min, and 2 = 10 min.

### Dimensional and visual analyses

All the 720 instruments included in the study were derived from a dimensionally compliant lot by the manufacturer (MicroMega, Besançon, France). However, ISO Standard 3630-1 allows for a certain range of dimensional tolerance, meaning that slight variations in dimension are considered acceptable and within compliance.

However, geometrical variations among endodontic instruments significantly influence their mechanical behavior. Slight differences in tip diameter or taper can directly impact bending and torsional test results [55]. Therefore, a dimensional analysis was performed on each of the 720 instruments using a 54× magnification profilometer and 1 µm resolution (MicroVu Sol161, MCE metrology, Paris, France) with the Calc-V3 software (version 1.16.1, MCE metrology, Paris, France). To avoid statistical bias linked to this variable, all statistical analyses incorporated the taper variations observed across the different instruments.

Taper measurements were conducted in accordance with ISO 3630-1 and calculated based on diameter measurements taken at 3 and 10 mm from the instrument tip. A total of 16 taper values were recorded, ranging from 4.86 to 7.14.

For the detection of corrosion on instruments following immersion in the irrigating solutions, visual assessments were conducted under double-blind conditions, with a magnification of ×54 and 1 µm resolution.

### Experimental procedure

Five instruments from each subgroup were immersed in 20 mL of the irrigation solution being tested. Borosilicate glass containers were used due to their resistance to chemical attack [56, 57].

The samples tested at 21 °C were prepared in the controlled environment of the technical hall of dental engineering, compliant with ISO 13485 standards. Irrigation solution tested at 35 °C was heated using an incubator (Firlabo TR5, Meyzieu, France). At the end of each immersion period, instruments were immediately washed with distilled water and dried with air to eliminate all residual chemical interactions, and then visually analyzed using the profilometer to detect signs of corrosion. After visual inspection, the instruments were subjected to bending or torsion tests in accordance with ISO 3630-1, using a custom-built torsion-bending test bench.

#### ISO 3630-1 bending test (45°) (Fig. 1)

Following calibration of the test bench, the instrument was carefully positioned against the bottom of the chuck and perpendicular to the device axis, ensuring the tip was not compressed. The first 3 mm of the instrument tip were embedded. Then, the assembly was tightened using a torque screwdriver at 65 N·cm. Finally, the test was launched with the bending arm positioned 18 mm from the tip.

**Fig. 1:**
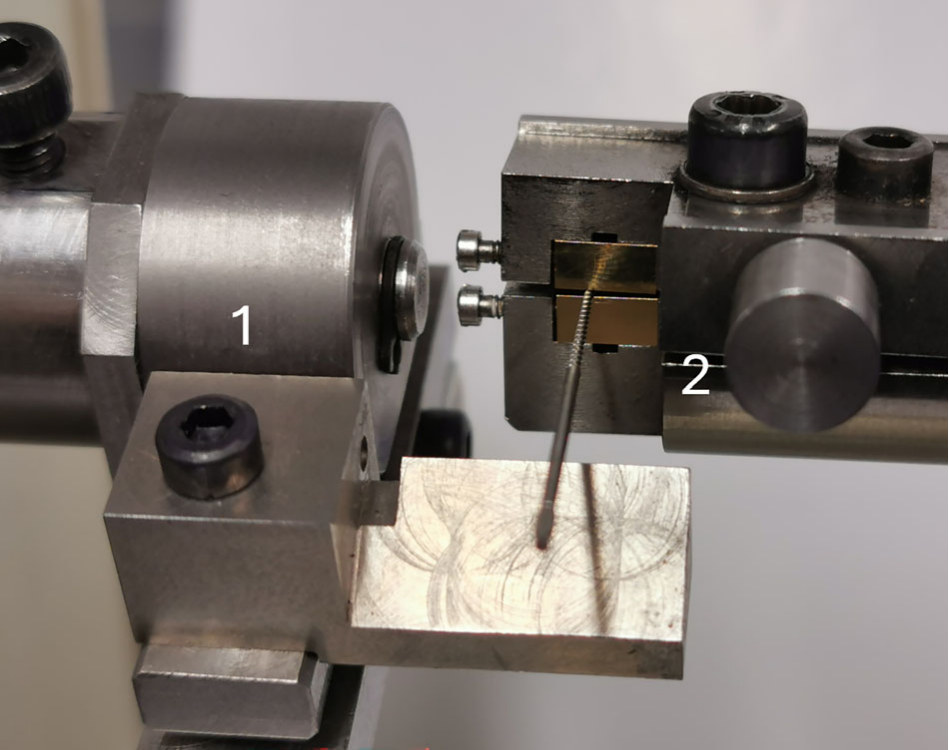
Bending test setup. The instrument tip was embedded over the first 3 millimeters, and the bending point was located 18 millimeters from the tip. 1: test bench motor; 2: test bench sensor.

The maximum torque was recorded during a 45° flexion.

#### ISO 3630-1 torsional fracture test (Fig. 2)

Following calibration of the test bench, the instrument was carefully inserted against the bottom of the chuck to embed the first 3 mm of the instrument tip, ensuring the tip was not compressed. Then, the assembly was tightened using a torque screwdriver at 65 N·cm. Then, the chuck screw on the non-cutting side of the instrument was loosened, and a spacer was inserted between the chucks. Once contact was made on both sides using the handwheel, the instrument was locked with the torque screwdriver, the spacer was removed, and the torsional test was initiated until instrument fracture occurred.

**Fig. 2:**
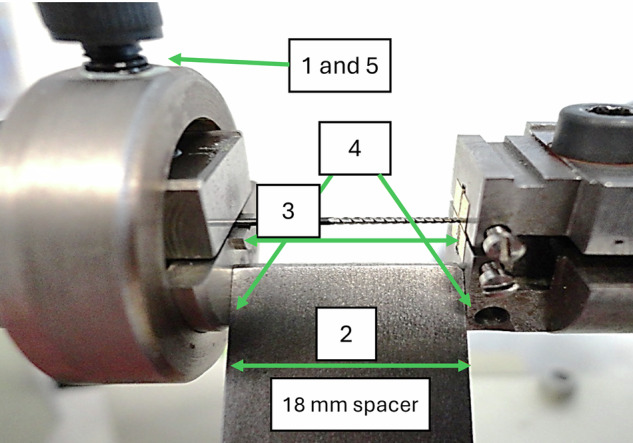
Torsional test setup. After the tip was embedded: 1. Loosen the screw on the non-cutting side of the instrument; 2. Insert the spacer; 3. Validate the position of the instrument; 4. Block the spacer; 5. Lock the setup with the torque screwdriver and remove the spacer.

The maximum torque and angle at fracture were recorded.

### Statistics

We investigate the influence of experimental conditions—comprising 4 irrigation solutions, 2 temperatures, 3 heat treatments, and 3 exposure times, resulting in 72 distinct settings. For each, only 5 observations are available. Our goal is to quantify the effect of these settings on mechanical measurements, including flexion (maximum torque) and torsion (maximum deflection angle and maximum fracture torque).

To make efficient and robust use of the limited data, we employ median regression with categorical covariates representing the settings. Since the taper of the instrument may also influence the mechanical measurements [55], we include it as an additional covariate. As taper is continuous, care must be taken to model it without introducing mis-specification.

Two extremes would be (i) assuming a linear, setting-independent contribution and (ii) discretizing taper and modeling full interactions with all settings. The latter, while well specified, introduces too many parameters for reliable estimation. Thus, we consider the following five models:

- Model 1: Response ∼ Irrigation Solutions×Temperature×Heat Treatment×Exposure Time (baseline, no taper effect).
- Model 2: as in Model 1, with an additive linear effect of taper shared across all settings.
- Model 3: as in Model 1, with an additive non-linear effect of taper, modeled using its three terciles.
- Model 4: as in Model 1, with a linear interaction between taper and all categorical variables (taper effects vary across settings).
- Model 5: as in Model 1, with a non-linear interaction between taper and all settings.

To estimate median (Response | Setting, Taper), we use leave-one-out cross validation (LOOCV), in which each observation is held out in turn, the model is fit to the remaining data, and prediction error is evaluated on the held-out point. We use pseudo-R², the fraction of loss explained compared to the best constant predictor, to assess model fit.

#### Model comparison

Table 2 presents the LOOCV pseudo-R² values for each model across the three responses:

**Table 2:** LOOCV pseudo-R2 for each model and response.

Model	Max torque	Max deflection angle	Max fracture torque
Model 1	0.4770	0.3538	-0.0443
Model 2	0.4745	0.3520	-0.0437
Model 3	0.4885	0.3522	-0.0347
Model 4	0.1678	0.1245	-0.4322
Model 5	0.4074	0.2753	-0.1466

Model 3 performs best for both maximum torque and maximum fracture torque, while Model 1 performs best for maximum fracture angle. We adopt these models for subsequent causal inference of setting effects, adjusting for taper. Note that for the third response, maximum fracture torque, the pseudo-R² values are negative, reflecting a widespread lack of causal effect mixed with noise-induced estimation error. We deem the model still suitable for analysis, as our goal is to analyze the significance of the setting effects overall and relative to each other, not to optimize prediction accuracy.

#### Model diagnostics

**Fig. 3:**
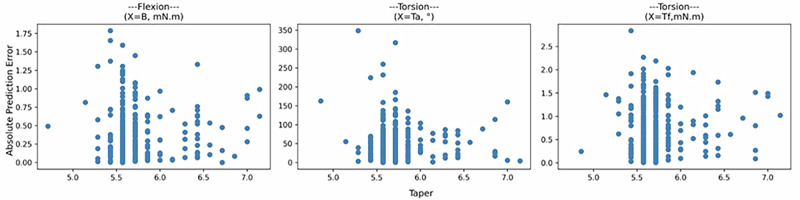
LOOCV loss as a function of taper for each response.

To assess model specification, we visualize the LOOCV loss as a function of taper (Fig. 3). Although taper is theoretically a continuous variable, only 16 distinct values are observed in the data, with several appearing multiple times. This results in vertical striping artifacts in the scatterplots due to the discreteness and repetition of values. These artifacts are not indicative of modeling issues but rather reflect the limited granularity of the observed taper distribution. Importantly, we do not observe any clear trend in the loss as a function of taper, such as increasing or decreasing error, which would suggest a mis-specified functional form. Given the absence of systematic patterns or strong residual trends in loss across taper values, we conclude that the selected models are sufficiently well specified to proceed with inference.

#### Hypothesis testing

To evaluate whether certain experimental settings have a statistically greater influence on the responses, we conduct pairwise tests *via* percentile bootstrapping.

Specifically, we consider:

- Test 1: whether different irrigation solutions yield significantly different responses, controlling for all other settings.
- Test 2: whether different heat treatments yield significantly different responses, controlling for all other settings.

This leads to:

$$2\times \left(\left(\begin{array}{c}4\\ 2\end{array}\right)\times 2\times 3\times 3+4\times 2\times \left(\begin{array}{c}3\\ 2\end{array}\right)\times 3\right)=360\,{pairwise\; tests}$$

We control the family-wise error rate (FWER) at α = 0.05 using the Holm-Bonferroni correction, a step-down improvement over the classic Bonferroni method. The procedure sorts the empirical *p*-values in increasing order and compares the smallest to $\frac{\alpha }{m}$, the next smallest to $\frac{\alpha }{m-1}$, and so on, where $m$ = 360 is the total number of tests. This yields a sequence of progressively less stringent rejection thresholds, improving statistical power while still rigorously controlling the FWER.

As we rely on empirical p-values derived *via* percentile bootstrapping, their minimum granularity is $\frac{1}{{{{{\rm{n}}}}}_{{{{\rm{bootstraps}}}}}}$. To ensure sufficient resolution for detection under the Holm procedure, we choose:

$${{{{\rm{n}}}}}_{{{{\rm{bootstraps}}}}}=\frac{20}{{{{\rm{mini}}}}\left(\frac{{{{\rm{\alpha }}}}}{{{{\rm{m}}}}-{{{\rm{i}}}}+1}\right)}=144000$$

This guarantees that even the smallest threshold (associated with the first ordered test) can be reached. Note that when a p-value is observed as 0 due to bootstrap resolution, we interpret it as being at most $\frac{1}{{{{{\rm{n}}}}}_{{{{\rm{bootstraps}}}}}}\approx 6.94\times {10}^{-6}$, which may still lead to rejection under the Holm-Bonferroni threshold sequence.

Ethical approval and consent were not required for this study on dental instruments.

## Results

### Influence of the irrigation solutions on mechanical performance of NiTi endodontic files

The objective of the first test was to evaluate the influence of irrigation solutions on the mechanical behavior of endodontic instruments by comparing the mechanical performance of instruments exposed to different irrigation solutions under identical experimental conditions. The X(a,b,c,d) groups were tested in bending and torsion, while the temperature (b), heat treatment (c), and exposure time (d) variables were kept constant. Only the irrigation solution variable (a) was modified.

**Fig. 4:**
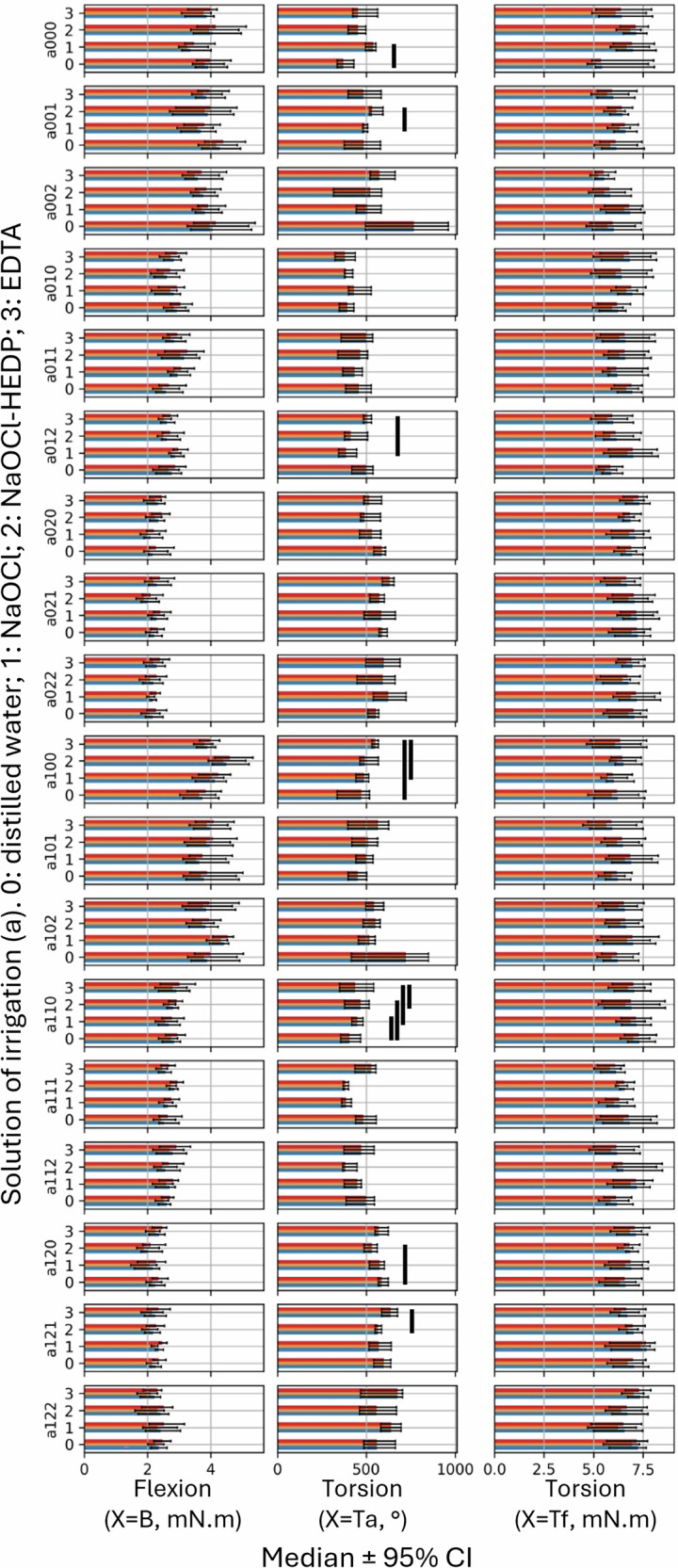
Predicted medians with 95% CI for the distinct irrigation solutions in an otherwise identical setting. The x-axis represents the predicted median ± 95% CI for each type of performed test (X). The y-axis shows the different groups following the formula X (a,b,c,d), where a varies according to the different irrigating solutions tested. Each histogram consists of the three taper terciles (C1 in blue, C2 in yellow, and C3 in red). Black bars indicate statistical significance at *P* < 0.0001.

For X = B, no significant differences in maximum bending torque were observed under any of the tested conditions (Fig. 4). Under the experimental conditions of our study, canal irrigants did not affect the bending mechanical behavior of the endodontic instruments.

For X = Tf, no significant differences in maximum torsional torque at fracture were observed under any of the tested conditions (Fig. 4). Under the experimental conditions of this study, canal irrigants did not affect the torsional mechanical behavior at fracture of endodontic instruments.

For X = Ta, 11 significant differences were observed regarding the maximum angular deflection at torsional fracture (Fig. 4). EDTA was the irrigation solution most frequently associated with an increase of maximum torsional angular deflection of endodontic instruments, followed by distilled water. NaOCl resulted in the lowest torsional angular deflection, followed by NaOCl-HEDP.

### Influence of heat treatment on mechanical performance of NiTi endodontic files after exposition in different irrigation solution

The objective of the second test was to evaluate the influence of heat treatment on the mechanical behavior of endodontic instruments after exposition in different irrigation solution. The X(a,b,c,d) groups were tested in bending and torsion, while the irrigation solution (a), temperature (b), and exposure time (d) variables were kept constant. Only the heat treatment variable (c) was modified.

**Fig. 5:**
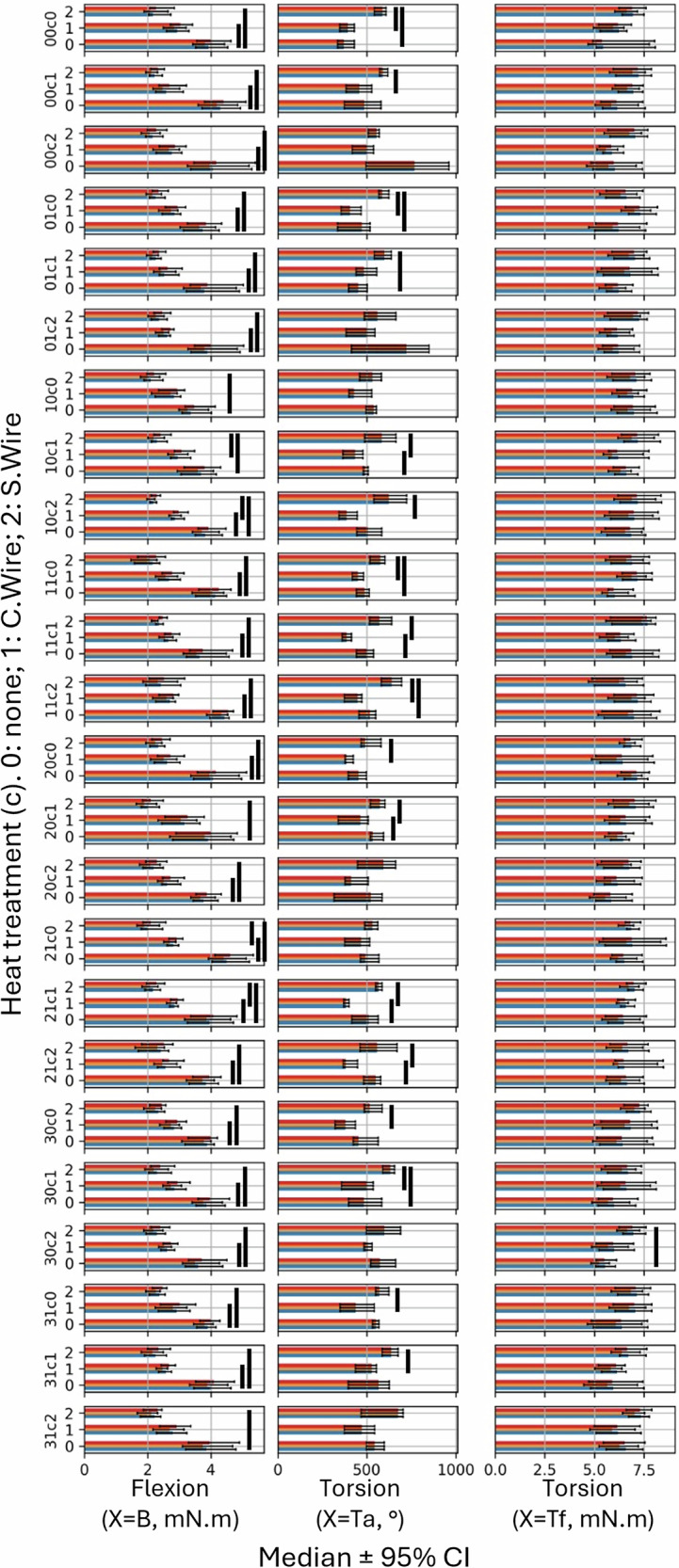
Predicted medians with 95% CI for the distinct heat treatments in an otherwise identical setting. The x-axis represents the predicted median ± 95% CI for each type of performed test (X). The y-axis shows the different groups following the formula X(a,b,c,d), where c varies according to the different heat treatment tested. Each histogram consists of the three taper terciles (C1 in blue, C2 in yellow, and C3 in red). Black bars indicate statistical significance at *P* < 0.0001.

For X = B, 48 significant differences were observed regarding the maximum bending torque (Fig. 5). The results show superior bending mechanical behavior for S.Wire instruments, followed by C.Wire instruments, regardless of solution irrigation, temperature and exposure time conditions.

For X = Tf, 1 significant difference was observed regarding the maximum torsional torque at fracture (Fig. 5). S.Wire instruments appear to exhibit greater resistance to torsional fracture.

For X = Ta, 27 significant differences were observed regarding the maximum angular deflection at fracture (Fig. 5). S.Wire instruments showed the highest torsional fracture angles, followed by non-heat-treated instruments, regardless of the irrigation solution, temperature and exposure time conditions.

### Visual analysis

**Fig. 6:**
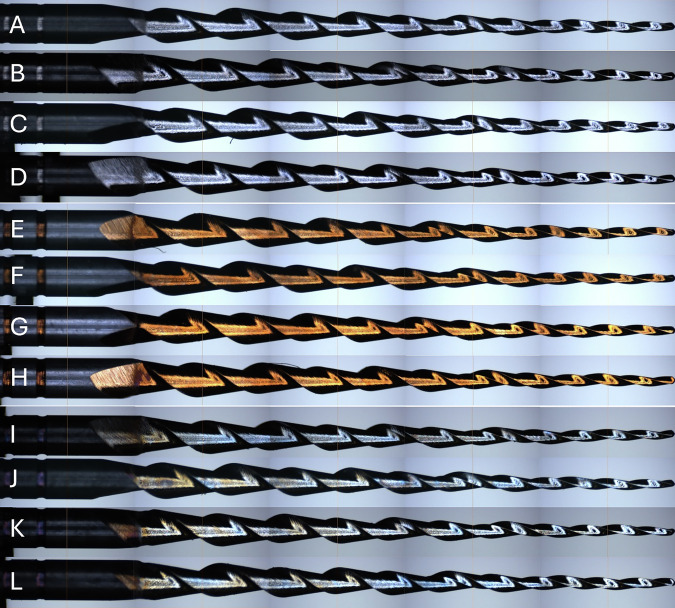
Visual analysis of endodontic files after exposure to the different irrigation solutions. **A**–**D**: visual analysis of non-heat-treated endodontic files after exposure to distilled water (**A**), 3% NaOCl (**B**), NaOCl-HEDP (**C**), and 17% EDTA (**D**) for 10 min at 35 °C. E to H: visual analysis of C.Wire endodontic files after exposure to distilled water (**E**), 3% NaOCl (**F**), NaOCl-HEDP (**G**), and 17% EDTA (**H**) for 10 min at 35 °C. I to L: visual analysis of S.Wire endodontic files after exposure to distilled water (**I**), 3% NaOCl (**J**), NaOCl-HEDP (**K**), and 17% EDTA (**L**) for 10 min at 35 °C.

After profilometric examination at 54× magnification, no signs of corrosion were observed under our experimental conditions (Fig. 6).

## Discussion

### Influence of the irrigation solutions on mechanical performance of NiTi endodontic files

Previous studies investigating the effects of NaOCl, EDTA, and HEDP on NiTi endodontic instruments have primarily focused on surface alterations and mechanical properties [29–52]. Several of these studies have reported corrosion, increased surface roughness, or reduced mechanical performance, particularly after prolonged exposure to NaOCl or EDTA under conditions that do not reflect typical clinical usage [31, 33, 34, 44]. However, a study observed no significant changes in mechanical resistance or corrosion even after several hours of immersion in NaOCl [42]. In the same way, the influence of temperature on irrigant-induced effects remains unclear. Berutti et al. [32] reported that short-term exposure to NaOCl at 50 °C led to increased corrosion and reduced fracture resistance [32]. In contrast, Cavalleri et al. [35] found no signs of corrosion under similar thermal and exposure conditions [35].

At room or body temperature, several investigations have demonstrated increased surface roughness or microstructural degradation of NiTi instruments following short-term immersion (<10 min) in NaOCl or EDTA [30, 36–38, 41]. These effects have been associated with reductions in cyclic fatigue resistance across various NiTi systems under both static and dynamic testing conditions [46–49]. Conversely, other studies at room or body temperature have concluded that brief exposure (<10 min) to NaOCl or EDTA does not significantly affect the cyclic fatigue resistance and does not cause any increase of fracture risk [43, 45, 50]. Similarly, each type of irrigant could influence the mechanical response of endodontic instruments differently. A study reported that immersion in NaOCl had no adverse effect on cyclic fatigue, whereas immersion in 17% EDTA for three minutes significantly reduced fatigue resistance.

Overall, the literature is inconclusive regarding the effects of irrigants on the structural and mechanical properties of endodontic instruments. One major contributing factor may be the wide variability in study protocols, specifically in terms of difference in tested instruments. Several authors have emphasized that the response of NiTi instruments to irrigants depends strongly on their specific manufacturing process, surface treatment, and heat treatment [34, 41, 46–48, 53]. Therefore, the mechanical behavior of NiTi files exposed to irrigants is likely influenced by the combination of the instrument’s metallurgical characteristics and the irrigant used.

To our knowledge, our study is the first to evaluate three instruments with identical geometrical design but differing thermal treatments, thereby allowing direct comparison under constant geometric conditions. Under our controlled and clinically relevant experimental conditions, no significant effect was observed on either maximum torque in torsion or bending for any of the irrigants tested. These findings support the hypothesis that short-term contact with irrigants under clinical conditions does not significantly compromise the mechanical integrity or surface characteristics of NiTi instruments.

Recent investigations have also examined the use of DualRinse HEDP, a combination of NaOCl and etidronic acid that enables simultaneous irrigation and chelation. Studies investigated the impact of NaOCl-HEDP on the mechanical performance of rotary and reciprocating NiTi instruments and reported a slight decrease in cyclic fatigue resistance compared to NaOCl alone [52, 53]. Again, the effect appeared to depend on variables such as exposure time, temperature, and instrument type. Our findings contribute to this discussion by demonstrating that, under clinically relevant conditions, NaOCl resulted in the lowest torsional angular deflection while NaOCl-HEDP produced slightly higher torsional angular deflection. Conversely, EDTA was the irrigation solution most frequently associated with an increase in the torsional angular deflection at fracture, followed by distilled water. This suggests that, under controlled conditions, NaOCl-HEDP exerts a milder effect on NiTi ductility compared to NaOCl alone, and a less favorable effect compared to EDTA. This intermediate behavior might be due to its less potent chelating action compared to EDTA and its stabilizing effect on NaOCl. These findings are of particular scientific relevance considering the expanding implementation of continuous chelation strategies in contemporary clinical endodontic protocols. However, further studies are needed to evaluate of DualRinse on different heat-treated NiTi systems.

### Influence of heat treatment on mechanical performance of NiTi endodontic files after exposition in different irrigation solution

The mechanical behavior of NiTi endodontic instruments is strongly influenced by their phase composition, which depends on the type of thermomechanical treatment applied during manufacturing. Conventional NiTi instruments, those without specific heat treatment, are predominantly in the austenitic phase at room and body temperature. These alloys are characterized by high stiffness, lower angular deflection at fracture, and reduced flexibility [58, 59]. In contrast, hybrid instruments such as those manufactured with C.Wire technology (e.g., One Curve) combine both austenitic and martensitic phases. This dual-phase structure leads to improved flexibility and greater resistance to cyclic fatigue compared to conventional austenitic NiTi. However, their mechanical properties remain inferior to those of fully martensitic alloys, especially in terms of angular deflection and elastic modulus [60].

Fully martensitic instruments, including those made from CM Wire, Blue, or S.Wire, exhibit the highest flexibility, greatest angular deflection at fracture, and superior resistance to cyclic fatigue. These improvements are due to the stable presence of the martensitic phase (or R-phase) at clinical temperatures, resulting in a lower elastic modulus and better mechanical adaptability to root canal curvature [60]. Interestingly, our results clearly highlighted that the C.Wire-treated instruments exhibited a lower deflection angle at fracture compared to instruments without thermal treatment. However, our findings showed no significant difference in torsional resistance between the untreated and the C.Wire-treated instruments. While the S.Wire instruments seem to demonstrate superior torsional resistance, our results are not consistent enough to draw a definitive conclusion.

Our study suggests that the mechanical advantages conferred by heat treatment are preserved regardless of the irrigant used, and that brief exposure to these solutions does not negatively impact the structural integrity or mechanical performance of NiTi endodontic instruments. In conclusion, short-term irrigant exposure does not compromise instrument safety. Unlike many prior studies, our protocol reflects the dynamic chemical and thermal environment of clinical endodontics, providing a more realistic assessment of instrument performance.

It should be noted as a limitation that only a single file brand and design (One Curve, MicroMega) was investigated. Consequently, the results may not be generalizable to all NiTi systems.

### Visual analysis

Many studies have pointed out the potential influence of galvanic corrosion due to electrochemical interaction between the NiTi working portion and the handle, especially in the presence of NaOCl [31, 34]. These studies showed that galvanic coupling could accelerate corrosion when the instrument’s handle is in contact with irrigants. However, in clinical practice, the handle remains outside the canal and is not exposed to irrigants.

In our study, the handles were not present, ensuring that any effects observed were due exclusively to the NiTi alloy itself and not to galvanic interactions. Our results show no signs of surface corrosion were detected on the NiTi instruments after exposure to any of the tested irrigants, as confirmed by profilometric analysis. This finding suggests that short-term contact with 3% NaOCl, 17% EDTA, or the NaOCl-HEDP combination under clinically relevant conditions does not induce measurable corrosive effects on the instrument surface. These results are in accordance with previous studies [35, 42] and seem to be in agreement with our mechanical results.

## Conclusion

(i) Our study contributes to a better understanding of the effects of irrigation solutions on the mechanical performance of NiTi endodontic instruments under clinically relevant conditions. Using three instruments with identical geometry but differing heat treatments, we found that short-term exposure to NaOCl, EDTA, or NaOCl-HEDP at room and intracanal temperature did not significantly alter the maximum torque or bending resistance of the instruments. However, variations in angular deflection at fracture were observed among irrigants, with EDTA associated with increased ductility, while NaOCl showed the lowest angular deflection. Interestingly, NaOCl-HEDP exhibited intermediate behavior, suggesting a milder effect on NiTi ductility, likely due to its reduced chelating strength and stabilizing action on NaOCl.

(ii) Our results confirm that heat treatment significantly enhances the mechanical performance of NiTi instruments, with S-Wire showing the highest flexibility and angular deflection, followed by C-Wire, and then non-heat-treated NiTi. These differences reflect their respective metallurgical structures: martensitic, hybrid, and austenitic. Importantly, exposure to NaOCl, EDTA, or NaOCl-HEDP under clinically relevant conditions did not compromise the mechanical behavior of any instrument type. This indicates that the beneficial effects of thermal treatment remain stable regardless of irrigant contact, and that short-term chemical exposure does not adversely affect the structural integrity or performance of NiTi endodontic files.

## Data Availability

The data that support the findings of this study are available from the corresponding author upon reasonable request.
